# Utilization of Sugarcane Bagasse by *Halogeometricum borinquense* Strain E3 for Biosynthesis of Poly(3-hydroxybutyrate-*co*-3-hydroxyvalerate)

**DOI:** 10.3390/bioengineering4020050

**Published:** 2017-05-25

**Authors:** Bhakti B. Salgaonkar, Judith M. Bragança

**Affiliations:** Department of Biological Sciences, Birla Institute of Technology and Science Pilani, K K Birla, Goa Campus, NH-17B, Zuarinagar, Goa 403 726, India; judith@goa.bits-pilani.ac.in

**Keywords:** archaea, halophiles, sugarcane bagasse, polyhydroxyalkanoates, *Halogeometricum borinquense* strain E3, Soxhlet extractor

## Abstract

Sugarcane bagasse (SCB), one of the major lignocellulosic agro-industrial waste products, was used as a substrate for biosynthesis of polyhydroxyalkanoates (PHA) by halophilic archaea. Among the various wild-type halophilic archaeal strains screened, *Halogeometricum borinquense* strain E3 showed better growth and PHA accumulation as compared to *Haloferaxvolcanii* strain BBK2, *Haloarcula japonica* strain BS2, and *Halococcus salifodinae* strain BK6. Growth kinetics and bioprocess parameters revealed the maximum PHA accumulated by strain E3 to be 50.4 ± 0.1 and 45.7 ± 0.19 (%) with specific productivity (qp) of 3.0 and 2.7 (mg/g/h) using NaCl synthetic medium supplemented with 25% and 50% SCB hydrolysate, respectively. PHAs synthesized by strain E3 were recovered in chloroform using a Soxhlet apparatus. Characterization of the polymer using crotonic acid assay, X-ray diffraction (XRD), differential scanning calorimeter (DSC), Fourier transform infrared (FT-IR), and proton nuclear magnetic resonance (^1^H-NMR) spectroscopy analysis revealed the polymer obtained from SCB hydrolysate to be a co-polymer of poly(3-hydroxybutyrate-*co*-3-hydroxyvalerate) [P(3HB-*co*-3HV)] comprising of 13.29 mol % 3HV units.

## 1. Introduction

Conventional plastics obtained from non-renewable petrochemical resources are creating environmental havoc due to their non-degradable nature. To solve this problem, various bio-based materials derived from renewable resources have been explored as a replacement for conventional plastics. These materials could be (i) directly extracted from biomass as polysaccharides, lignocelluloses, proteins, and lipids; (ii) chemically synthesized, e.g., by in vitro polymerization of bio-derived monomers such as lactate to produce poly(lactic acid) (PLA); or (iii) biologically synthesized by microorganisms, in vivo polymerization of hydroxylalkanoic acid (HA) units to polyhydroxyalkanoates (PHAs) [1]. PHAs are synthesized and accumulated as inclusions by microorganisms when the available nitrogen/phosphorus gets depleted while carbon is in excess. PHAs are synthesized either in the inner membrane, on a central scaffold, or in the cytoplasm of cells and aggregated in the form of globular, water-insoluble granules [2,3]. 

High production costs, downstream processing, and low yields are the major hurdles for the commercial production and application of PHA, making microbially synthesized PHA 5–10 times more expensive than petroleum-derived polymers [4]. Carbon sources/substrates represent half of the PHA fermentation cost [5,6,7]. Various strategies such as replacing commercial substrates with inexpensive renewable agro-industrial waste, finding novel high PHA-accumulating microorganisms or microbial strain improvements, and reducing the cost of PHA recovery/downstream process can make the overall fermentation process more cost-effective [4].

Sugarcane (*Saccharum officinarum*) is the world’s largest cash crop, with Brazil being the leading producer, followed by India, and is exploited for the production of sugar, jaggery, ethanol, molasses, alcoholic beverages (rum), soda, etc. [8]. Sugarcane bagasse (SCB) is the leftover, fibrous residue of sugarcane stalk after the extraction of juice and is a major lignocellulosic, inexpensive byproduct of the sugarcane industry [9]. SCB needs special attention for its management, and is primarily used as a source of energy for electricity/biogas production. It also serves as a raw material for fermentation processes in the production of various products such as enzymes (cellulose, lipase, xylanase, inulinase, and amylase), animal feed (single cell protein), amino acids, organic acids, bioethanol, bioplastics, etc. [10]. 

SCB is comprised of cellulose (46%), hemicelluloses (27%), lignin (23%), and ash (4%) [9]. Cellulose is a homopolysaccharide comprised of a linear chain of β(1→4) linked d-glucose units, whereas hemicellulose is a heteropolysaccharide consisting of many different sugar monomers such as xylan (consisting units of pentose sugar, xylose), glucuronoxylan (consisting units of glucuronic acid and xylose), arabinoxylan (consisting of copolymers of two pentose sugars, arabinose and xylose), glucomannan (consisting of d-mannose and d-glucose), and xyloglucan (consisting of units of glucose and xylose). Degradation of these polymers would yield various sugars that could serve as a substrate for PHA production. The fibrous nature of SCB makes its microbial degradation slower and more difficult and hence limits its utilization. To overcome this, pre-treatment of SCB is usually carried out for improved substrate availability and to speed up the fermentation process. Acid hydrolysis is a fast and simple method, mostly performed to release sugars from the SCB that can be readily used by microorganisms, rather than feeding solid bagasse, which is time-consuming and interferes with the downstream processing [11].

Much insight has been gained into PHA synthesis by using non-halophilic microorganisms as compared to their halophilic counterparts. PHA production by halophiles has been studied among members of the families *Halobacteriaceae* (Domain *Archaea*) and *Halomonadaceae* (Domain *Bacteria*). Kirk and Ginzburg (in 1972) first documented the occurrence of PHA granules in halophilic archaea [12]. Subsequent research on members of the family *Halobacteriaceae* revealed significant PHA synthesis by the following genera: *Haloferax* (*Hfx. mediterranei*), *Haloarcula* (*Har. marismortui*), *Halobacterium* (*Hbt. noricense*), *Haloterrigena* (*Htg. hispanica*), *Halogeometricum* (*Hgm. borinquense*), *Halococcus* (*Hcc. dombrowskii*), *Natrinema* (*Natrinema pallidum*); *Halobiforma* (*Hbf. haloterrestris*), and *Halopiger* (*Hpg. aswanensis*) [13,14,15,16,17,18,19,20]. Among the halophilic bacteria, members of the genus *Halomonas* (*H. boliviensis*) are known to synthesize a large amount of PHA from a variety of substrates such as glucose, maltose, starch hydrolysate, etc. [21,22]. 

Overall, there are few reports on PHA production using agro-industrial waste/cheap substrates by employing halophilic microorganisms. *Haloferax mediterranei* is the most widely studied and is reported to produce a copolymer of poly(3-hydroxybutyrate-*co*-3-hydroxyvalerate) [P(3HB-*co*-3HV)] using various renewable agro-industrial waste products like extruded corn starch, rice bran, wheat bran, hydrolyzed whey, waste stillage from the rice-based ethanol industry, vinasse, etc. [23,24,25,26]. Pramanik et al. and Taran reported the synthesis of a homopolymer of hydroxybutyrate (PHB) by *Haloarcula marismortui* MTCC 1596 and *Haloarcula* sp. IRU1 by utilization of vinasse waste from the ethanol industry and petrochemical wastewater, respectively [27,28].

To date, to the best of our knowledge, there has been no report on the utilization of sugarcane bagasse for PHA production by members of the domain Archaea, especially the genus *Halogeometricum*. Therefore, in the present study, the ability of halophilic archaeal isolates to accumulate PHA from SCB hydrolysate was examined. The growth kinetics and bioprocess parameters during growth and PHA production by *Hgm. borinquense* strain E3 were examined and the polymer synthesized was characterized using physico-chemical analysis techniques. 

## 2. Materials and Methods

### 2.1. HalophilicArchaeal Strains and Media Used

Four halophilic archaeal isolates (GenBank/DDBJ database accession number), *Halococcus salifodinae* strain BK6 (AB588757), *Haloferax volcanii* strain BBK2 (AB588756), and *Haloarcula japonica* strain BS2 (HQ455798), isolated from solar salterns of Ribandar in Goa, India, and *Hgm. borinquense* strain E3 (AB904833), obtained from Marakkanam in Tamil Nadu, India, were used in the present study [29,30]. The cultures were maintained on complex media (Table 1), i.e., NTYE (NaCl Tryptone Yeast Extract), NT (NaCl Tri-sodium citrate), EHM (Extremely Halophilic Medium). The NSM (NaCl Synthetic Medium) with a varying concentration of SCB hydrolysate as per the requirements was used as a production medium (Table 1).

### 2.2. Procurement, Processing, and Hydrolysis of Sugarcane Bagasse 

Sugarcane bagasse (SCB) was collected from a local sugarcane juice extractor, from Vasco-da-Gama, Goa, India. It was dried under sunlight for 3–5 days, cut into small ~5–10 cm pieces, followed by pulverization to a fine powder using a blender. The powdered form of the waste was subjected to dilute acid hydrolysis. Briefly, 5 gm of the SCB powder was added to 100 mL of 0.75% (*v*/*v*) sulfuric acid in water. The mixture was heated at 100 °C for 1 h under reflux using an allihn condenser. The hydrolysate was filtered using non-absorbent cotton to separate the solid residue from the liquid hydrolysate. The liquid hydrolysate was neutralized to pH 7.0–7.4 using NaOH, followed by sterilization at 121 °C for 10 min and storage at 4 °C [11].

### 2.3. Characterization of the SCB

The SCB powder was characterized for the following physical and chemical parameters. Total solids (TS) and volatile solids (VS) were estimated according to the American Public Health Association (APHA) [31]. The chemical oxygen demand (COD) was determined as described by Raposo et al. [32]. The carbon (C), hydrogen (H), nitrogen (N), and sulfur (S) contents of the SCB were determined using a CHNS Analyzer (Elementar, Rhine Main area near Frankfurt, Germany). The total carbohydrate content of the SCB hydrolysate was estimated by the phenol sulphuric acid method, as described by Dubois et al. [33]. Total Kjeldahl nitrogen (TKN) was determined as described by Labconco [34]. All the physical and chemical characterization was performed in triplicate to determine means and standard deviations.

### 2.4. Screening of the Halophilic Archaeal Isolates for PHA Accumulation using SCB Hydrolysate

Halophilic archaeal isolates were screened for the production of PHA using NSM (Table 1) and Nile Red stain. Briefly, the NSM plates were prepared by adding 1.8% agar (*w*/*v*) to the medium followed by autoclaving; while still molten, the medium was supplemented with varying concentrations (0.5%–30% *v*/*v*) of SCB hydrolysate along with 50 µL of Nile Red stain [stock of 0.01% (*w*/*v*) Nile red in DMSO] such that the final concentration was0.5 µg/mL medium. Twenty microliters of log phase (three-day-old) halophilic archaeal cultures were spot inoculated on the agar plates and incubated at 37 °C for 6–7 days. The plates were exposed to ultraviolet (UV) light using gel documentation system (BIO-RAD Laboratories, Hercules, CA, USA) andthe emitted fluorescence from the culture was quantified using TotalLab Quant software [16,35].

### 2.5. Selection and Further Study of the Best PHA Producer Strain

Based on it having the best growth and fluorescence on NSM supplemented with SCB hydrolysate, the halophilic archaeon *Hgm. borinquense* strain E3 was selected for further study. Preliminary screening indicted the strain E3 to grow up to 30% (*v*/*v*) of SCB hydrolysate. Therefore, the concentration of SCB hydrolysate that inhibited the growth of strain E3 was determined by growing the culture on NSM agar plates containing higher concentrations of the SCB hydrolysate, i.e., 50%, 75%, and 100%. NSM with 100% SCB hydrolysate was prepared by dissolving the medium ingredients in the directly SCB hydrolysate. Based on the growth observed on NSM agar plates, growth of the strain E3 was further recorded in NSM broth with that particular concentration of SCB hydrolysate. 

### 2.6. The Growth Kinetics and PHA Quantification

Growth and intracellular PHA content for *Hgm. borinquense* strain E3 were determined as follows. An actively growing mid-log phase culture (3–4 days) of *Hgm. borinquense* strain E3 grown in NGSM (NaCl Glucose Synthetic Medium) containing 0.2% glucose was used as a starter culture. One percent of the starter culture was inoculated in NSM (NaCl Synthetic Medium) containing 25% and 50% (*v*/*v*) SCB hydrolysate. The flasks were maintained at 37 °C, 110 rpm on a rotary shaker (Skylab Instruments, Mumbai, India). At regular intervals of 24 h, aliquots of the culture broth wereaseptically withdrawn and the following parameters were monitored: (i) culture growth was monitored by recording the absorbance at 600 nm using a UV-visible spectrophotometer (UV-2450, Shimadzu, Tokyo, Japan) with the respective medium as blank, (ii) Cell Dry Mass (CDM) was determined by centrifuging 2 mL of the culture broth at 12,000 *g* for 15 min, washing the pellet with distilled water, and recentrifuging it at 12,000 *g* for 15 min, followed by drying at 60 °C until a constant weight was obtained. Since the SCB hydrolysate had some particle participation, the dry mass of the plain medium (without culture) was taken and subtracted from the culture CDM so as to avoid error. (iii) Total carbohydrates were determined colorimetrically according to Dubios et al. and compared with the standard curve [33]; (iv) the pH of the medium was monitored using a pH meter; and (v) polymer quantification was done by converting PHA to crotonic acid using concentrated sulfuric acid. The absorbance was recorded at 235 nm using a UV-visible spectrophotometer (UV-2450, Shimadzu, Tokyo, Japan) and compared with the standard curve for PHB [36]. All the experiments were performed in triplicate to determine means and standard deviations.

### 2.7. Extraction of the PHA

The PHA extraction from the biomass was done as described by Sánchez et al. with slight modifications [37]. Briefly, *Hgm. borinquense* strain E3 was grown in NSM containing 25% SCB hydrolysate for six days. The cells were harvested by centrifuging at 12,000 *g* for 10 min using Eppendorf centrifuge 5810R (Hamburg, Germany). The cell pellet was dried for 12 h at 60 °C in an oven (Bio Technics, Mumbai, India). The dried cells were ground using mortar and pestle and extracted for 8–10 h at 60 °C in a soxhlet extractor using chloroform. Up to 95% of the chloroform was recollected using rotary evaporator (Rotavapor R-210, Büchi, Switzerland) and the remaining 5% of the chloroform-containing polymer was poured into a clean glass Petri dish and kept undisturbed during the total evaporation of the chloroform to give a uniform polymer film. 

### 2.8. Characterization of the PHA

Characterization of the polymer was done using UV-visible spectrophotometry (crotonic acid assay), X-ray diffraction (XRD) analysis, differential scanning calorimeter (DSC) analysis, Fourier transform infrared (FT-IR) spectroscopy, and proton nuclear magnetic resonance (^1^H-NMR) spectroscopy, as described in detail by Salgaonkar and Bragança [30].

## 3. Results and Discussion

### 3.1. Sugarcane Bagasse (SCB)

The SCB used in the present study appeared greenish-brown with a sweet odor and upon pulverization had total solids (TS) of 94.3 ± 0.14%, volatile solids (VS) of 92.7 ± 0.14%, and COD of 1.18 ± 0.05 g/Kg. The carbon (C), hydrogen (H), nitrogen (N), and sulfur (S) content of the SCB was found to be C = 43.45 ± 0.12%, H = 6.0 ± 0.27%, N = 0.26 ± 0.02%, and S = 0.32 ± 0.12%. Hydrolysis of SCB using dilute sulfuric acid yielded total carbohydrates of 12.64 ± 0.7 g/L and total Kjeldahl nitrogen (TKN) of 0.7 g/L (Table S1).

### 3.2. Screening for PHA using SCB Hydrolysate

All four halophilic archaeal isolates were able to grow on NSM plates with Nile Red dye supplemented with SCB hydrolysate as substrate. Upon exposure of the plates to UV light, only three cultures, *Hfx. volcanii* strain BBK2, *Har. japonica* strain BS2, and *Hgm. borinquense* strain E3, showed bright orange fluorescence, indicating the accumulation of PHA (Figure S1). *Hcc. salifodinae* strain BK6 showed weak growth but failed to show any fluorescence. The intensity of fluorescence exhibited by the cultures varied, in the order *Hgm. borinquense* strain E3 > *Har. japonica* strain BS2 > *Hfx. volcanii* strain BBK2. *Hgm. borinquense* strain E3 grew faster and showed better fluorescence, which directly correlates withthe amount of polymer accumulated over a range of SCB concentrations (Figure S1). Preliminary work on *Hgm. borinquense* strain E3 proved it to be the best accumulator of PHA in an NGM medium supplemented with 2% glucose [30].

### 3.3. Optimization of SCB Hydrolysate Concentration

**Figure 1:**
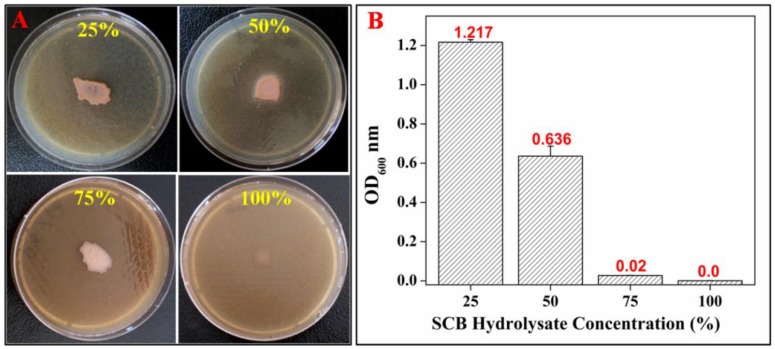
Growth of *Hgm*. *borinquense* strain E3 on (**A**) NSM agar plates and (**B**) NSM broth, containing various concentrations of SCB hydrolysate.

Figure 1 represents the growth of *Hgm*. *borinquense* strain E3 on NSM agar plates and broth containing various concentrations of SCB hydrolysate. The SCB hydrolysate optimization studies revealed that the culture could tolerate and grew up to 75% (*v*/*v*) SCB hydrolysate on NSM agar plates (Figure 1A). Interestingly, when grown in NSM broth (Figure 1B), the culture grew only up to 50% (*v*/*v*) SCB hydrolysate and failed to grow at higher concentrations. 

### 3.4. Growth Profile of Hgm. borinquense Strain E3 and Polymer Quantification Study

**Figure 2:**
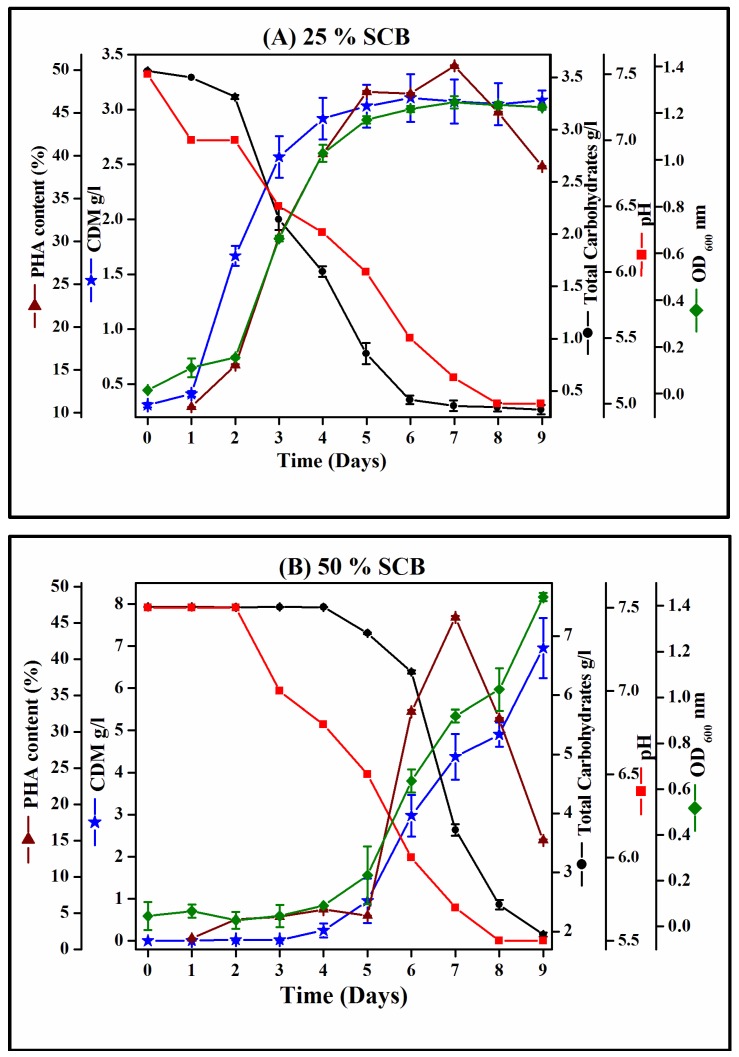
Growth profile and PHA production by *Hgm*. *borinquense* strain E3 in NSM containing (**A**) 25% and (**B**) 50% SCB hydrolysate.

The time course of growth of *Hgm. borinquense* strain E3 in NSM containing 25% and 50% SCB hydrolysate is presented in Figure 2. Table 2 gives a comparison of the various kinetics and bioprocess parameters used to determine growth and PHA production by *Hgm. borinquense* strain E3 using SCB hydrolysate. In the presence of 25% SCB hydrolysate, isolate E3 showed a 48-h lag and reached 3.17 ± 0.19 g/L of maximum cell dry mass (CDM), containing 1.6 ± 0.09 g/L of PHA. In the presence of 50% SCB hydrolysate, isolate E3 exhibited a longer lag phase of 96 h and reached 4.15 ± 0.7 g/L of maximum CDM, containing 1.9 ± 0.3 g/L of PHA. The lag phase of the culture depends on the definite environmental conditions. This prolonged lag phase could be reduced by increasing the inoculum size and decreasing the effect of culture conditions on the growth, which can be achieved by acclimatizing the starter culture to the ingredients of production medium by pre-culturing the isolate in the presence of the respective substrates concentration. The substantial quantity of carbohydrates, i.e., 12.64 ± 0.7 g/L in the SCB hydrolysate, served as the basic essential carbon source required for the growth and synthesis of PHA by *Hgm. borinquense* strain E3. The rapid consumption of the total carbohydrates by the isolate was observed as the growth progressed and a steady drop in the pH of the production medium from 7.2 to 5.0 was also noted.

To the best of our knowledge, haloarchaea have not been explored for their potential to utilize SCB hydrolysate for the synthesis of PHA. Bioprocess parameters (Table 2) revealed the maximum PHA accumulation by *Hgm. borinquense* strain E3 to be 50.4 ± 0.1 and 45.7 ± 0.19 (%) with specific productivity (qp) of 3.0 and 2.7 (mg/g/h) using NaCl synthetic medium supplemented with 25% and 50% SCB hydrolysate, respectively. A recent investigation by Pramanik et al. reported the potential of haloarchaeon *Har. marismortui* MTCC 1596 to produce 23 ± 1.0 and 30 ± 0.3 (%) P(3HB) with specific productivity (qp) of 1.21 and 1.39 (mg/g/h) using a nutrient-deficient medium (NDM) supplemented with 10% and 100% raw and treated vinasse, respectively [27]. The specific productivity (qp) attained by *Hgm. borinquense* strain E3 using SCB hydrolysate was higher compared to *Har. marismortui* MTCC 1596 grown in the presence of vinasse. Silva et al. (2004) investigated the synthesis of P(3HB) by *Burkholderia sacchari* IPT 101 and *Burkholderia cepacia* IPT 048 by feeding the cultures with SCB hydrolysate as a carbon source. It was noted that both the cultures reached 4.4 g/L of dry biomass, containing 62% and 53% of P(3HB) in the case of *B. sacchari* IPT 101 and *B. cepacia* IPT 048, respectively. The specific production rate and yield coefficient of the PHA were 0.11 (g/L/h) and 0.39 (g/g) for *B. sacchari* IPT 101, whereas it was 0.09 (g/L/h) and 0.29 (g/g) for *B. cepacia* IPT 048 [7].

Attempts have been made to reduce the fermentation cost of PHA by employing various haloarchaeal strains and examining their ability to utilize inexpensive substrates. Danis et al. showed the ability of *Natrinema pallidum*1KYS1 to produce 0.075, 0.055, 0.091, 0.039, 0.077, and 0.464 g/L of polymer by utilizing various waste products such as corn starch, sucrose, whey, melon, apple, and tomato as carbon substrates [18]. Pramanik et al. studied the ability of *Har. marismortui* to utilize 10% raw vinasse and 100% pre-treated vinasse to produce 2.8 g/L and 4.5 g/L of PHB [27]. Similarly, Bhattacharyya et al. employed *Hfx. mediterranei* to produce 19.7 g/L and 17.4 g/L from 25% and 50% pre-treated vinasse, respectively [26]. Also, 24.2 g/L PHBV biosynthesis was observed in *Hfx. mediterranei* with extruded cornstarch [23].

### 3.5. Bench Scale Polymer Production and Extraction by Hgm. borinquense Strain E3

The polymer was extracted from the cell biomass as shown in Figure S2. The dried cells of *Hgm. borinquense* strain E3 (before Soxhlet extraction), when subjected to concentrated H_2_SO_4_ hydrolysis, showed a clear peak at 235 nm, which is indicative of crotonic acid, indicating the presence of PHA (Figure S3A) [36]. After Soxhlet extraction, no peak at 235 nm was observed, thus confirming the complete extraction of the polymer from the cell mass (Figure S3B). 

### 3.6. Polymer Characterization

The polymer obtained using SCB hydrolysate (Figure S2J) appeared orange due to the co-extraction of carotenoid pigment along with some cellular lipids from the cells of *Hgm. borinquense* strain E3. These impurities were taken care of by treating the polymer with acetone for 10 min [30]. This polymer was characterized using a UV-visible spectrophotometer, XRD, DSC, FT-IR, and NMR analysis. 

#### 3.6.1. UV-Visible Spectrophotometric Analysis

Concentrated H_2_SO_4_ hydrolysis of the polymer obtained from SCB gave a characteristic peak at 235 nm of crotonic acid, which corresponded with the standard PHB (Sigma-Aldrich, St. Louis, MO, USA) (inset of Figure S3) and also with the copolymer P(3HB-*co*-3HV) synthesized by *Hgm. borinquense* strain E3 and *Hfx. mediterranei* “DSM 1411”when fed with substrates such as glucose and raw vinasse, respectively [30,26]. 

#### 3.6.2. XRD Analysis

**Figure 3:**
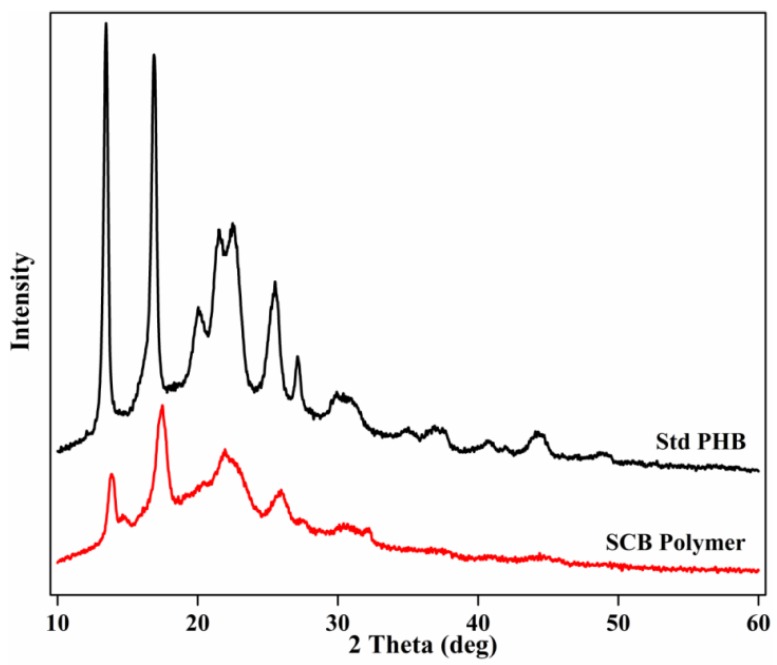
Comparison of X-ray diffraction patterns of standard PHB and polymer obtained from *Hgm. borinquense* strain E3 grown in NSM containing SCB hydrolysate.

Figure 3 represents the X-ray diffraction (XRD) patterns of the polymer obtained from SCB hydrolysate in comparison with standard PHB (Sigma-Aldrich, St. Louis, MO, USA). The profile of polymer from SCB exhibited prominent peaks at 2*θ* = 13.8°, 17.4°, 21.8°, 25.8°, and 30.7°, corresponding to (020), (110), (101), (121), and (002) reflections of the orthorhombic crystalline lattice. Overall, the diffraction pattern was similar to that of the PHB. However, peak shifts as well as a decrease in peak intensity were observed in the case of the SCB polymer when compared with standard PHB. It was clearly observed that the diffraction peaks between 2*θ* = 0–25° were broadened and drastically decreased in intensity for the SCB polymer. A broadening of the peaks indicates a decrease in crystallinity, i.e., the amorphous nature of the polymer [40]. The crystallite size L (nm) was determined for the highest peaks using the Scherrer equation, which is defined as: L(nm) = 0.94 *λ*/*B*Cos*θ*, where *λ* is the wavelength of the X-ray radiation, which is 1.542 Ǻ (wavelength of the Cu); *B* is the full width at half maximum (FWHM) in radians; and *θ* is the Bragg angle [41,42]. The crystallite size for the highest peak (020) in the case of standard PHB was found to be 22.3 nm, which decreased drastically to 10.4 nm for peak (110) of the SCB polymer. For the diffraction peak of the (110) plane of SCB polymer, an increase in the FWHM was observed as compared to the standard PHB. This clearly indicates a decrease in the crystallite size, given that the peak width is inversely proportional to the crystallite size. The crystallite size matched more or less to the one reported for the copolymer P(3HB-*co*-3HV) synthesized by *Hgm. borinquense* strain E3 by utilizing glucose, which was found to be 12.17 nm for the (110) peak [30].

#### 3.6.3. DSC Analysis

**Figure 4:**
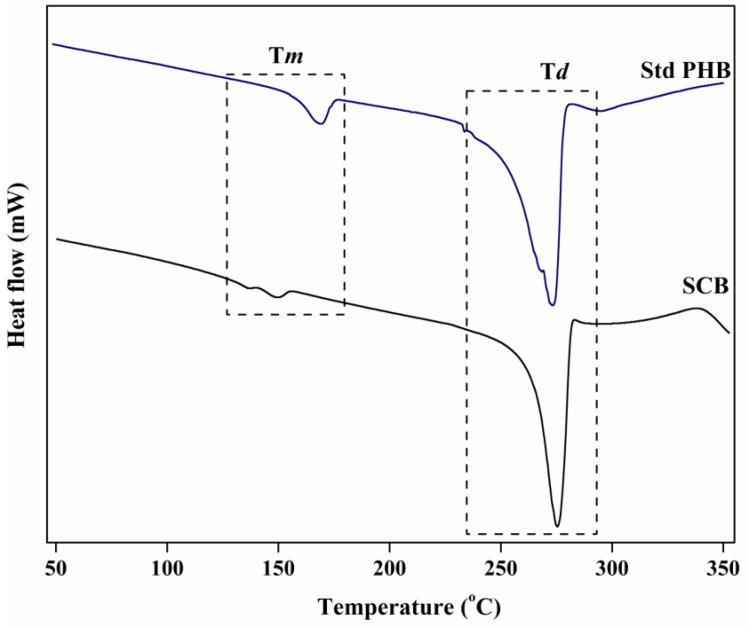
Comparison of DSC curves of standard PHB and polymer obtained from *Hgm. borinquense* strain E3 grown in NSM containing SCB hydrolysate.

The thermograms derived from differential scanning calorimetry (DSC) analysis for the polymer obtained using SCB hydrolysate and standard PHB (Sigma Aldrich, St. Louis, MO, USA) are represented in Figure 4. The polymer obtained from SCB hydrolysate exhibited two melting endotherms at T*m*_1_ = 136.5 °C and T*m*_2_ = 149.4 °C, whereas standard PHB displayed a single melting endotherm at T*m* = 169.2 °C. The degradation temperature (T*d*) peaks for the SCB polymer and PHB were at 275.4 °C and 273.2 °C, respectively (Table 3). A recent study by Buzarovska et al. (2009) reported two melting endotherms for pure copolymer PHBV containing 13 mol% 3-hydroxyvalerate (3HV) [43]. The lower melting peak (T*m*_1_) at ~138 °C could be due to the melting of the primary formed crystallites, whereas the upper one (T*m*_2_) at 152 °C is mostly due to the recrystallization of species during the scan [43]. The existence of multiple melting peaks in a polymer indicates that the polymers have varying monomer units such as 3HB and 3HV units [44]. *Haloferax mediterranei* is known to produce PHA with multiple melting endotherms by utilizing various carbon substrates [45]. Chen et al.(2006) and Koller et al. (2007)showed the ability of *Haloferax mediterranei* ATCC 33500/DSM 1411 to utilize extruded cornstarch/whey sugars as carbon substrates for the production of copolymer P(3HB-*co*-3HV) containing 10.4 mol% and 6 mol% of 3-hydroxyvalerate (3HV), respectively. The P(3HB-*co*-3HV) produced by strain DSM 1411 showed two melting peaks at 150.8 °C (T*m*_1_) and 158.9 °C (T*m*_2_), whereas the melting endotherms for strain ATCC 33500 were at 129.1 °C (T*m*_1_) and 144.0 °C (T*m*_2_) [13,46].

#### 3.6.4. FT-IR Analysis

**Figure 5:**
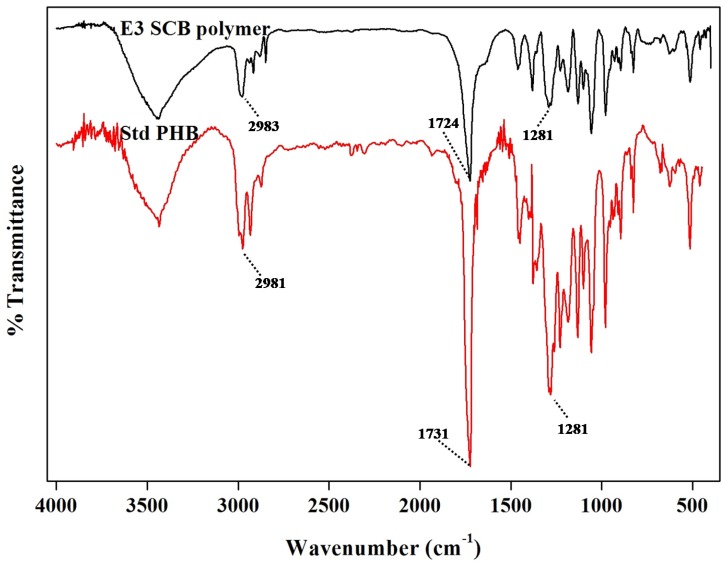
FT-IR spectra of standard PHB and polymer obtained from *Hgm. borinquense* strain E3 grown in NSM containing SCB hydrolysate.

The FT-IR spectra of polymer obtained using SCB hydrolysate were compared with those of the standard PHB (Sigma Aldrich, St. Louis, MO, USA) (Figure 5). The IR spectra of polymer obtained from SCB and standard PHB exhibited one intense absorption band at 1724 cm^−1^ and 1731 cm^−1^, respectively, characteristic of ester carbonyl group (C=O) stretching. A band at 1281 cm^−1^ represents C–O–C stretching, whereas one in the region 3100–2800 cm^−1^, i.e., 2983 cm^−1^ and 2981 cm^−1^, represents C–H stretching (Figure 5). Apart from these, other prominent bands were also observed, which may be due to interactions between the OH and C=O groups resulting in a shift of the stretching [40]. The peaks obtained for a polymer obtained from SCB hydrolysate matched well with those of the standard polymer.

#### 3.6.5. ^1^H-NMR Analysis

**Figure 6:**
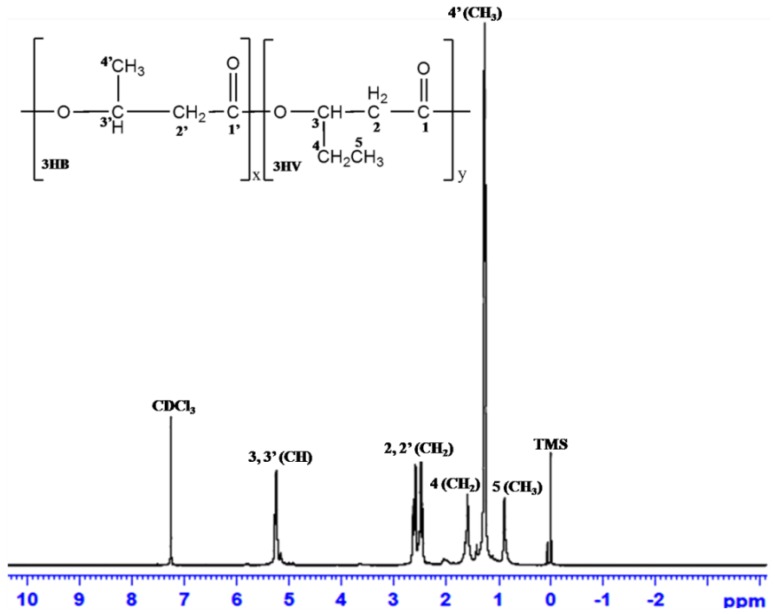
^1^H-NMR spectra of polymer from *Hgm. borinquense*strain E3 grown in NSM containing SCB hydrolysate.

^1^H-NMR scans of the polymer obtained from *Hgm*. *borinquense* strain E3 using SCB hydrolysate are represented in Figure 6. The chemical shift of the peaks and their chemical structure are represented in Table 4. Characteristic peaks at 0.889 ppm and 1.26 ppm are of methyl (CH_3_) from 3-hydroxyvalerate (3HV) and 3-hydroxybutyrate (3HB) unit, respectively. Therefore, it can be confirmed that the polymer obtained using SCB hydrolysate is a co-polymer of Poly(3-hydroxybutyrate-*co*-3-hydroxyvalerate) [P(3HB-*co*-3HV)]. The signals obtained from ^1^H-NMR correlated with those reported by Bhattacharyya et al. (2012) and Chen et al. (2006) for a co-polymer P(3HB-*co*-3HV)] obtained from *Hfx. mediterranei* strain DSM 1411 and strain ATCC 33500 by utilization of molasses spent wash (vinasse) and cornstarch, respectively (Figure 6, Table 4) [13,26]. Moreover, the ^1^H NMR spectrum of homopolymer of 3HB (P3HB) showed only one prominent peak at 1.25 ppm of methyl (CH_3_) from HB unit [35]. The co-polymer of P(3HB-*co*-3HV) comprised of 13.29% 3HV units, which was calculated as described by Salgaonkar and Bragança [30]. Interestingly, copolymer P(3HB-*co*-3HV)] containing a higher amount of 3HV (21.47% 3HV) was synthesized by the same E3 strain in NSM media with glucose as the substrate. The drastic reduction in 3HV units from 21.47% (glucose) to 13.29% (SCB) could be due to the inhibition of propionyl-coenzyme A synthesis, an important precursor of 3HV monomer by various byproducts of the SCB hydrolysate.

PHA accumulation by extremely halophilic archaea and moderately halophilic and/or halotolerant bacteria, inhabiting hypersaline and marine regions of countries such as China, Turkey, Bolivia, Vietnam, India, etc., has been documented [6,16,18,21,22]. Moderately halophilic bacteria belonging to the genus *Halomonas* such as *H*. *boliviensis* LC1, *H*. *nitroreducens*, and *H*. *salina* have been reported to accumulate 56.0%, 33.0%, and 55.0% (*w*/*w*) CDM of homopolymer of 3-hydroxybutyrate (3HB), i.e., P(3HB) by utilizing versatile substrates such as starch hydrolysate, glucose, and glycerol, respectively [22,47]. Similarly, Van-Thuoc et al. (2012) reported the ability of halophilic and halotolerant bacteria *Bacillus* sp. ND153 and *Yangia pacifica* QN271 to accumulate P(3HB) (65.0 and 48.0% *w*/*w* CDM)/PHBV (71.0 and 31.0% *w*/*w* of CDM) when glucose with or without propionate was provided as the carbon source [48]. However, there are very few reports on halophilic bacteria such as *H*. *campisalis* MCM B-1027 and *Yangia pacifica* ND199/ND218 synthesizing copolymer PHBV, irrespective of precursors like propionic/valeric acid in the culture medium [49,50]. Shrivastav et al.(2010) reported the utilization of *Jatropha* biodiesel byproduct as a substrate by *Bacillus sonorensis* strain SM-P-1S and *Halomonas hydrothermalis* strain SM-P-3M for the production of 71.8 and 75.0% (*w*/*w*) CDM of P(3HB), respectively [50].

Various members of halophilic archaea, belonging to the family *Halobacteriaceae*, such as *Halopiger aswanensis* strain 56 and *Hgm. borinquense* strain TN9 have been reported to accumulate 34.0% and 14.0% (*w*/*w*) CDM of homopolymer of P(3HB) by utilizing versatile substrates such as glucose, yeast extract, butyric acid, and sodium acetate [16,20]. Interestingly, *Hfx*. mediterranei is known to accumulate 23.0% (*w*/*w*) CDM of copolymer PHBV from glucose naturally, without any addition of precursor [23]. There are limited reports on the utilization of SCB hydrolysates as substrates for PHA production by microorganisms. Silva et al. reported the ability of two Gram-negative soil bacteria, *Burkholderia sacchari* IPT 101 and *Burkholderia cepacia* IPT 048, to accumulate poly-3-hydroxybutyrate (P3HB) when cultivated in SCB hydrolysate by submerged fermentation (SMF) [7]. 

Yu and Stahl reported the ability of the Gram-negative bacterium *Ralstonia eutropha* to synthesize both P(3HB) and P(3HB-*co*-3HV) when grown on SCB hydrolysate along with glucose as a carbon substrate. However, the bacterium failed to synthesize the polymer when grown in a hydrolysate solution devoid of glucose and was also unable to utilize pentose sugars like xylose and arabinose as a sole source of carbon [51]. Interestingly, in the present study, isolate *Hgm. borinquense* strain E3 was able to grow and synthesize PHA [P(3HB-*co*-3HV)] from crude SCB hydrolysate without any supplementation of carbon substrate (glucose) or prior treatment of the SCB hydrolysate for the removal of inhibitors. The strain E3 was also able to utilize arabinose and xylose when supplied as the sole source of carbon. Further studies should be done to investigate the cell and polymer yield after pre-treatment of the SCB hydrolysate to remove toxic substances. Also, the effect of glucose or other carbon substrates as supplements to SCB hydrolysate could be investigated with respect to increasing the PHA yield. 

## 4. Conclusions

In the present study, NSM with crude SCB hydrolysate was used as a carbon substrate for the production of PHA by the extremely halophilic archaeon, *Hgm. borinquense* strain E3. The maximum PHA accumulation was observed on the seventh day, reaching a total dry biomass of 3.17 and 4.15 g/L, containing 50.4% and 45.7% PHA. The polymer exhibited two melting endotherms and was identified to be a co-polymer of P(3HB-*co*-3HV) comprising of 13.29% 3HV. Strain E3 accumulated a substantial quantity of PHA using crude SCB hydrolysate without any prior treatment or additional carbon substrate. Investigation into enhancing the quality and yield of P(3HB-*co*-3HV) from SCB hydrolysate could be achieved by: (i) standardization of the production medium, by additional supplement of carbon substrate such as glucose/xylose, and (ii) detoxification/pre-treatment of SCB hydrolysate for removal of inhibitors. Reducing the long lag phase of the culture by increasing the inoculum concentrations and optimization of other cultivation parameters such as pH, temperature, aeration, or salt can further increase the biomass and polymer yield.

The potential application of *Hgm. borinquense* strain E3 for the utilization of agro-industrial waste such as SCB has been clearly demonstrated in the present study. Since India’s economy is dominated by agriculture and agro-based industries, large amounts of agro-industrial waste are being generated. Various agro-industrial waste products that can be degraded by such halophilic microbes should be explored for the production of biopolymers as this may help with both managing waste and cutting down the costs of commercial substrates. PHAs from halophilic archaea can be looked upon as a promising prospect for exploring novel bioplastics. This polymer can be further studied for various medical applications like tissue engineering and as a scaffold in organ culture. 

## Figures and Tables

**Table 1:** Composition of maintenance and production media used in the study.

Ingredients (g/L)	Maintenance Media			Production Medium
	NTYE	NT	EHM	NSM
NaCl	250.0	250.0	250.0	200.0
MgSO_4_·7H_2_O	20.0	20.0	20.0	-
MgCl_2_·6H_2_O	-	-	-	13.0
KCl	5.0	2.0	2.0	4.0
Tryptone	5.0	-	-	-
Yeast Extract	3.0	10.0	10.0	1.0
Tri-Sodium citrate	-	3.0	-	-
CaCl_2_·2H_2_O	-	-	0.36	1.0
NaBr			0.23	-
NaHCO_3_	-	-	0.06	0.2
NH_4_Cl	-	-	-	0.2
KH_2_PO_4_	-	-	-	0.5
Peptone	-	-	5.0	-
FeCl_3_·6H_2_O	-	-	Trace	0.005

NTYE: NaCl Tryptone Yeast Extract; NT: NaCl Tri-sodium citrate; EHM: Extremely Halophilic Medium; NSM: NaCl Synthetic Medium. NSM with various concentration of SCB was used as production media. Agar 1.8% (*w*/*v*) was use as solidifying agent. The pH of the medium was adjusted to 7.0–7.2 using 1M NaOH.

**Table 2:** Kinetic and bioprocess parameters during growth and PHA production by *Hgm. borinquense* strain E3 (present study) in comparison with *Har. marismortui* MTCC 1596 (literature).

HalophilicArchaeal Strain	Production Medium	Lag (h)	CDM (g/L)	PHA (g/L)	PHA Content (%)	µmax (1/h)	qp ^a^ (mg/g/h)	Y_P/S _^b^	Vol. Productivity ^c^ (g/L/h)	Reference
*Hgm. borinquense* strain E3	NSM25% SCB	48	3.17 ± 0.19	1.6 ± 0.09	50.4 ± 0.1	0.017	3.0	0.448	0.0095	Present study
	NSM50% SCB	96	4.15 ± 0.7	1.9 ± 0.3	45.7 ± 0.19	0.023	2.7	0.253	0.0113	
	NGSM2% Glucose	-	5.78 ± 0.4	4.25 ± 0.045	73.5 ± 0.045	ND	4.3	0.212	0.0252	[30] *
*Har. marismortui* MTCC 1596	NDM10% Raw vinasse	96 ± 12	12.0 ± 0.20	2.8 ± 0.2	23 ± 1.0	0.086	1.21	2.17	0.015	[27]
	NDM100% treated vinasse	144 ± 12	15.0 ± 0.35	4.5 ± 0.2	30 ± 0.3	0.128	1.39	0.77	0.020	

NSM: NaCl Synthetic Medium; NGSM: NaCl Glucose Synthetic Medium; NDM: Nutrient Deficient Medium; ND: not determined; μmax: maximum specific growth rate; CDM: Cell Dry Mass; PHA: Polyhydroxyalkanoate; *Hgm*: *Halogeometricum*; *Har*: *Haloarcula*; MTCC: Microbial Type Culture Collection; ^a^ Specific production rate of PHA (qp) = PHA (g/L)/time (h) × CDM (g/L) [38]; ^b^ Yield coefficient of PHA (Y_P/S_) = PHA (g/L)/ total organic carbon (g/L). The Y_P/S_ was calculated based on total carbohydrate in NSM with 25% (3.5642 g/L) and 50% SCB hydrolysate (7.494 g/L) [26]; ^c^ Volume productivity of PHA = PHA (g/L)/ time (h) [39]. Time of growth for *Hgm. borinquense* strain E3 using NSM supplemented with glucose and SCB hydrolysate was 168 h (7 days), whereas that for *Har. marismortui* MTCC 1596 using NDM and 10% raw and 100% treated vinasse was 192 h (8 days) and 216 h (9 days), respectively [27]; * Some of the bioprocess parameters are not mentioned in the reference.

**Table 3:** Comparison of the DSC data of a polymer synthesized by *Hgm*. *borinquense* strain E3 using SCB waste with data from the literature.

PHA from Various Substrates	Haloarchaeal Isolate	DSC Characterization (°C)			Reference
		T*m*1	T*m*2	T*d*	
SCB	*Hgm. borinquense* strain E3	136.59	149.4	275.4	Present study
Glucose		138.15	154.74	231.08	[30]
Cornstarch	*Hfx. mediterraneic* ATCC 33500	129.1	144.0	NR	[13]
Whey	*Hfx. mediterranei* DSM 1411	150.8	158.9	241	[46]

T*m*—melting temperature; T*d*—degradation temperature NR-not reported

**Table 4:** Comparison of the chemical shift of the peaks and their chemical structure, obtained from ^1^H-NMR data of polymer synthesized by *Hgm*. *borinquense* strain E3 using SCB waste with data from the literature.

PHA from Various Substrates	Haloarchaeal Isolate	Relative Chemical Structure					Reference
		CH_3_ (3HB)	CH_2_ (3HV/3HB)	CH (3HV/3HB)	CH_3_ (3HV)	CH_2_ (3HV)	
		Chemical Shifts of Each Peak (ppm)					
SCB	*Hgm. borinquense* strain E3	1.26–1.27	2.44–2.63	5.22–5.27	0.889	1.618–1.635	Present study
Glucose		1.26–1.28	2.44–2.63	5.26	0.85–0.91	1.6	[30]
Cornstarch	*Hfx. mediterranei* ATCC 33500	1.2	2.5	5.2	0.9	1.6	[13]
Vinasse	*Hfx. mediterranei* DSM 1411	1.26–1.28	2.43-2.645	5.22-5.28	0.86–0.95	1.586	[26]

HB: hydroxybutyrate; HV: hydroxyvalerate; CH_3_: methyl; CH_2_: methylene; CH: methane.

## Supplementary Materials

The supplementary materials are available online at http://www.mdpi.com/2306-5354/4/2/50/s1.

## Abbreviations

APHA	American Public Health Association
CDM	cell dry mass
COD	chemical oxygen demand
DSC	Differential scanning calorimetry
EHM	Extremely Halophilic Medium
FT-IR	Fourier transform infrared
NMR	nuclear magnetic resonance
Har.	*Haloarcula*
Hbf.	*Halobiforma*
Hbt.	*Halobacterium*
Hcc.	*Halococcus*
Hfx.	*Haloferax*
Hgm.	*Halogeometricum*
Hpg.	*Halopiger*
Htg.	*Haloterrigena*
Nnm.	*Natrinema*
NSM	NaCl synthetic medium
NTYE	NaCl Tryptone Yeast extract
P(3HB-*co*-3HV)	poly(3-hydroxybutyrate-*co*-3-hydroxyvalerate)
P3HB	Poly-3-hydroxybutyrate
PHA	Polyhydroxyalkanoates
SCB	sugarcane bagasse
TKN	Total Kjeldahl nitrogen
TS	total solids
VS	volatile solids
XRD	X-ray diffraction

## References

[1] Chen G.Q., Patel M.K. (2011). Plastics derived from biological sources: Present and future: A technical and environmental review. Chem. Rev..

[2] Jendrossek D., Pfeiffer D. (2014). New insights in the formation of polyhydroxyalkanoate granules (carbonosomes) and novel functions of poly(3-hydroxybutyrate). Environ. Microbiol..

[3] Vadlja D., Koller M., Novak M., Braunegg G., Horvat P. (2016). Footprint area analysis of binary imaged *Cupriavidus necator* cells to study PHB production at balanced, transient, and limited growth conditions in a cascade process. Appl. Microb. Biotechnol..

[4] Koller M., Maršálek L., de Sousa Dias M.M., Braunegg G. (2017). Producing microbial polyhydroxyalkanoate (PHA) biopolyesters in a sustainable manner. New Biotechnol..

[5] Valappil S.P., Boccaccini A.R., Bucke C., Roy I. (2007). Polyhydroxyalkanoates in Gram-positive bacteria: Insights from the genera *Bacillus* and *Streptomyces*. Antonie Leeuwenhoek..

[6] Han J., Hou J., Liu H., Cai S., Feng B., Zhou J., Xiang H. (2010). Wide distribution among halophilic archaea of a novel polyhydroxyalkanoate synthase subtype with homology to bacterial type III synthases. Appl. Environ. Microbiol..

[7] Silva L.F., Taciro M.K., Ramos M.M., Carter J.M., Pradella J.G.C., Gomez J.G.C. (2004). Poly-3-hydroxybutyrate (P3HB) production by bacteria from xylose, glucose and sugarcane bagasse hydrolysate. J. Ind. Microbiol. Biotechnol..

[8] Parameswaran B. (2009). Sugarcane bagasse. Biotechnology for Agro-Industrial Residues Utilisation.

[9] Pippo W.A., Luengo C.A. (2013). Sugarcane energy use: Accounting of feedstock energy considering current agro-industrial trends and their feasibility. Int. J. Energy Environ. Eng..

[10] Obruca S., Benesova P., Marsalek L., Marova I. (2015). Use of lignocellulosic materials for PHA production. Chem. Biochem. Eng. Q..

[11] Lavarack B.P., Griffin G.J., Rodman D. (2002). The acid hydrolysis of sugarcane bagasse hemicellulose to produce xylose, arabinose, glucose and other products. Biomass Bioenergy.

[12] Kirk R.G., Ginzburg M. (1972). Ultrastructure of two species of halobacterium. J. Ultrastruct. Res..

[13] Chen C.W., Don T.M., Yen H.F. (2006). Enzymatic extruded starch as a carbon source for the production of poly(3-hydroxybutyrate-*co*-3-hydroxyvalerate) by *Haloferax mediterranei*. Process Biochem..

[14] Han J., Lu Q., Zhou L., Zhou J., Xiang H. (2007). Molecular characterization of the phaECHm genes, required for biosynthesis of poly(3-hydroxybutyrate) in the extremely halophilicarchaeon *Haloarculamarismortui*. Appl. Environ. Microbiol..

[15] Romano I., Poli A., Finore I., Huertas F.J., Gambacorta A., Pelliccione S., Nicolaus G., Lama L., Nicolaus B. (2007). *Haloterrigena hispanica* sp. nov., an extremely halophilicarchaeon from Fuente de Piedra, Southern Spain. Int. J. Syst. Evol. Microbiol..

[16] Salgaonkar B.B., Mani K., Bragança J.M. (2013). Accumulation of polyhydroxyalkanoates by halophilic archaea isolated from traditional solar salterns of India. Extremophiles.

[17] Legat A., Gruber C., Zangger K., Wanner G., Stan-Lotter H. (2010). Identification of polyhydroxyalkanoates in *Halococcus* and other haloarchaeal species. Appl. Microbiol. Biotechnol..

[18] Danis O., Ogan A., Tatlican P., Attar A., Cakmakci E., Mertoglu B., Birbir M. (2015). Preparation of poly(3-hydroxybutyrate-*co*-hydroxyvalerate) films from halophilic archaea and their potential use in drug delivery. Extremophiles.

[19] Hezayen F.F., Rehm B.H.A., Eberhardt R., Steinbuchel A. (2000). Polymer production by two newly isolated extremely halophilic archaea: Application of a novel corrosion-resistant bioreactor. Appl. Microbiol. Biotechnol..

[20] Hezayen F.F., Gutierrez M.C., Steinbuchel A., Tindall B.J., Rehm B.H.A. (2010). *Halopiger aswanensis* sp. nov., a polymerproducing and extremely halophilicarchaeon isolated from hypersaline soil. Int. J. Syst. Evol. Microbiol..

[21] Guzmán H., Van-Thuoc D., Martín J., Hatti-Kaul R., Quillaguamán J. (2009). A process for the production of ectoine and poly(3-hydroxybutyrate) by *Halomonas boliviensis*. Appl. Microbiol. Biotechnol..

[22] Quillaguaman J., Hashim S., Bento F., Mattiasson B., Hatti-Kaul R. (2005). Poly(β-hydroxybutyrate) production by a moderate halophile, *Halomonas boliviensis* LC1 using starch hydrolysate as substrate. J. Appl. Microbiol..

[23] Huang T.Y., Duan K.J., Huang S.Y., Chen C.W. (2006). Production of polyhydroxyalkanoates from inexpensive extruded rice bran and starch by *Haloferax mediterranei*. J. Ind. Microbiol. Biotechnol..

[24] Koller M., Hesse P., Bona R., Kutschera C., Atlić A., Braunegg G. (2007). Potential of various archae-and eubacterial strains as industrial polyhydroxyalkanoate producers from whey. Macromol. Biosci..

[25] Bhattacharyya A., Saha J., Haldar S., Bhowmic A., Mukhopadhyay U.K., Mukherjee J. (2014). Production of poly-3-(hydroxybutyrate-*co*-hydroxyvalerate) by *Haloferax mediterranei* using rice-based ethanol stillage with simultaneous recovery and re-use of medium salts. Extremophiles.

[26] Bhattacharyya A., Pramanik A., Maji S.K., Haldar S., Mukhopadhyay U.K., Mukherjee J. (2012). Utilization of vinasse for production of poly-3-(hydroxybutyrate-*co*-hydroxyvalerate) by *Haloferax mediterranei*. AMB Express.

[27] Pramanik A., Mitra A., Arumugam M., Bhattacharyya A., Sadhukhan S., Ray A., Mukherjee J. (2012). Utilization of vinasse for the production of polyhydroxybutyrate by *Haloarcula marismortui*. Folia Microbiol..

[28] Taran M. (2011). Utilization of petrochemical wastewater for the production of poly(3-hydroxybutyrate) by *Haloarcula* sp. IRU1. J. Hazard. Mater..

[29] Mani K., Salgaonkar B.B., Bragança J.M. (2012). Culturable halophilic archaea at the initial and final stages of salt production in a natural solar saltern of Goa, India. Aquat. Biosyst..

[30] Salgaonkar B.B., Bragança J.M. (2015). Biosynthesis of poly(3-hydroxybutyrate-*co*-3-hydroxyvalerate) by *Halogeometricumborinquense* strain E3. Int. J. Biol. Macromol..

[31] American Public Health Association, American Water Works Association (1981). Standard Methods for the Examination of Water and Wastewater: Selected Analytical Methods Approved and Cited by the United States Environmental Protection Agency.

[32] Raposo F., De la Rubia M.A., Borja R., Alaiz M. (2008). Assessment of a modified and optimised method for determining chemical oxygen demand of solid substrates and solutions with high suspended solid content. Talanta.

[33] Dubois M., Gilles K.A., Hamilton J.K., Rebers P.A.T., Smith F. (1956). Colorimetric method for determination of sugars and related substances. Anal. Chem..

[34] Labconco C. (1998). A Guide to Kjeldahl Nitrogen Determination Methods and Apparatus.

[35] Salgaonkar B.B., Mani K., Braganca J.M. (2013). Characterization of polyhydroxyalkanoates accumulated by a moderately halophilic salt pan isolate *Bacillus megaterium* strain H16. J. Appl. Microbiol..

[36] Law J.H., Slepecky R.A. (1961). Assay of poly-β-hydroxybutyric acid. J. Bacteriol..

[37] Sánchez R.J., Schripsema J., da Silva L.F., Taciro M.K., Pradella J.G., Gomez J.G.C. (2003). Medium-chain-length polyhydroxyalkanoic acids (PHA mcl) produced by Pseudomonas putida IPT 046 from renewable sources. Eur. Polym. J..

[38] Follonier S., Panke S., Zinn M. (2011). A reduction in growth rate of *Pseudomonas putida* KT2442 counteracts productivity advances in medium-chain-length polyhydroxyalkanoate production from gluconate. Microb. Cell Factories.

[39] Castilho L.R., Mitchell D.A., Freire D.M. (2009). Production of polyhydroxyalkanoates (PHAs) from waste materials and by-products by submerged and solid-state fermentation. Bioresour. Technol..

[40] Da Silva Pinto C.E., Arizaga G.G.C., Wypych F., Ramos L.P., Satyanarayana K.G. (2009). Studies of the effect of molding pressure and incorporation of sugarcane bagasse fibers on the structure and properties of poly (hydroxy butyrate). Compos. Part A Appl. Sci. Manuf..

[41] Vidhate S., Innocentini-Mei L., D’Souza N.A. (2012). Mechanical and electrical multifunctional poly(3-hydroxybutyrate-*co*-3-hydroxyvalerate)-multiwall carbon nanotube nanocomposites. Polym. Eng. Sci..

[42] Oliveira L.M., Araújo E.S., Guedes S.M.L. (2006). Gamma irradiation effects on poly(hydroxybutyrate). Polym. Degrad. Stab..

[43] Buzarovska A., Grozdanov A., Avella M., Gentile G., Errico M. (2009). Poly(hydroxybutyrate-*co*-hydroxyvalerate)/titanium dioxide nanocomposites: A degradation study. J. Appl. Polym. Sci..

[44] Sudesh K. (2012). Polyhydroxyalkanoates from Palm Oil: Biodegradable Plastics.

[45] Hermann-Krauss C., Koller M., Muhr A., Fasl H., Stelzer F., Braunegg G. (2013). Archaeal production of polyhydroxyalkanoate (PHA) co-and terpolyesters from biodiesel industry-derived by-products. Archaea.

[46] Koller M., Hesse P., Bona R., Kutschera C., Atlić A., Braunegg G. (2007). Biosynthesis of high quality polyhydroxyalkanoate co-and terpolyesters for potential medical application by the archaeon *Haloferax mediterranei*. Macromol. Symp..

[47] Cervantes-Uc J.M., Catzin J., Vargas I., Herrera-Kao W., Moguel F., Ramirez E., Lizama-Uc G. (2014). Biosynthesis and characterization of polyhydroxyalkanoates produced by an extreme halophilic bacterium, *Halomonas nitroreducens*, isolated from hypersaline ponds. J. Appl. Microbiol..

[48] Van-Thuoc D., Huu-Phong T., Thi-Binh N., Thi-Tho N., Minh-Lam D., Quillaguaman J. (2012). Polyester production by halophilic and halotolerant bacterial strains obtained from mangrove soil samples located in Northern Vietnam. Microbiol. Open.

[49] Kulkarni S.O., Kanekar P.P., Nilegaonkar S.S., Sarnaik S.S., Jog J.P. (2010). Production and characterization of a biodegradable poly(hydroxybutyrate-*co*-hydroxyvalerate) (PHB-*co*-PHV) copolymer by moderately haloalkalitolerant *Halomonas campisalis* MCM B-1027 isolated from Lonar Lake, India. Bioresour. Technol..

[50] Shrivastav A., Mishra S.K., Shethia B., Pancha I., Jain D., Mishra S. (2010). Isolation of promising bacterial strains from soil and marine environment for polyhydroxyalkanoates (PHAs) production utilizing Jatropha biodiesel byproduct. Int. J. Biol. Macromol..

[51] Yu J., Stahl H. (2008). Microbial utilization and biopolyester synthesis of bagasse hydrolysates. Bioresour. Technol..

