# Supplementary material for: Utilization of Sugarcane Bagasse by Halogeometricum borinquense Strain E3 for Biosynthesis of Poly(3-hydroxybutyrate-co-3-hydroxyvalerate)
Source: Bioengineering (Basel). 2017 May 25;4(2):50. doi: 10.3390/bioengineering4020050 (PMC5590456; doi:10.3390/bioengineering4020050)
Supplement: Supplementary file 1 [file bioengineering-04-00050-s001.pdf]

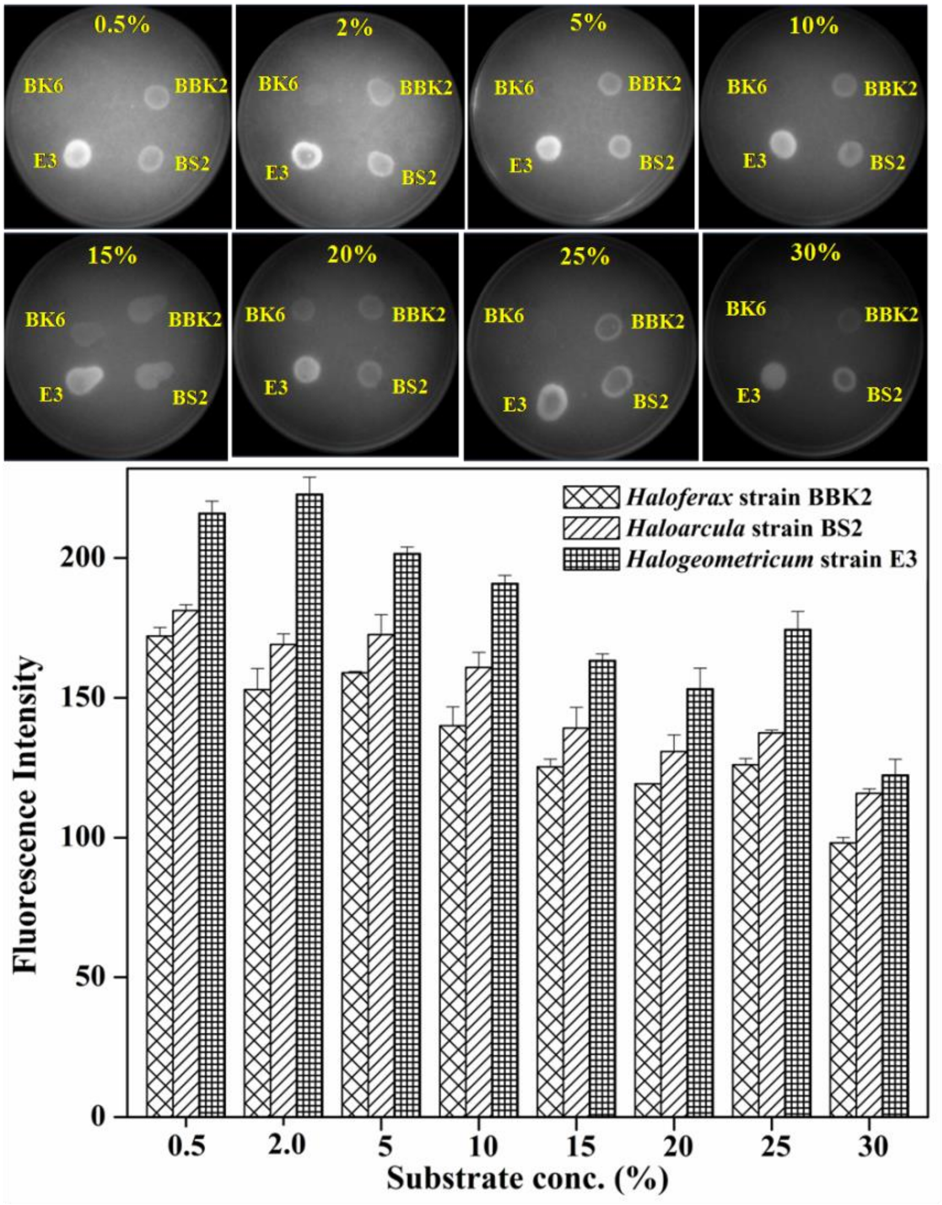

Figure S1. Fluorescence exhibited by the PHA accumulating haloarchaeal cultures, grown on NSM agar plates containing Nile Red dye and various concentrations (%) of SCB hydrolysate. The fluorescence emitted after UV exposure was quantified and compared using TotalLab Quant software.

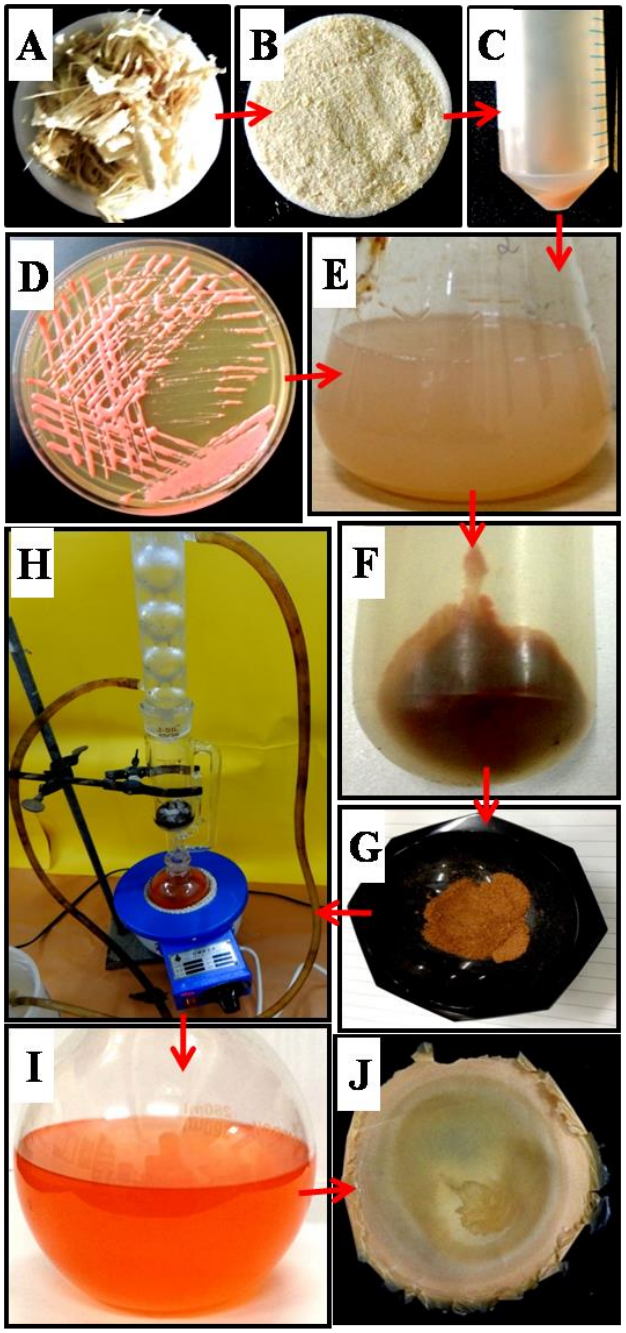

Figure S2. Schematic illustration of the production and extraction of PHA from *Hgm. borinquense* strain E3 using SCB. (A) SCB fibers, (B) fine powder of pulverized SCB, (C) liquid hydrolysate obtained after dilute acid hydrolysis of SCB, (D) pure culture of *Hgm. borinquense* strain E3, (E) liquid culture grown in NSM containing SCB hydrolysate, (F) culture pellet containing PHA, (G) dry cell powder obtained after drying the cells containing PHA at 70 ℃ and grinding using mortar and pestle, (H) polymer extraction from the cells with chloroform using soxhlet apparatus, (I) chloroform containing the polymer and (J) polymer film obtained after solvent evaporation.

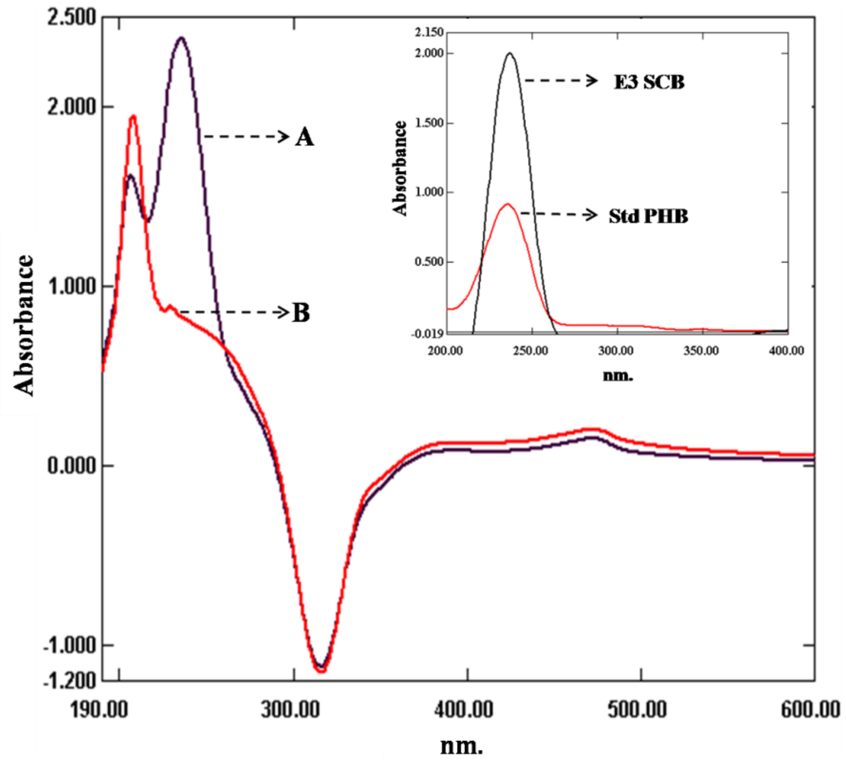

Figure S3. Spectrophotometric scans of concentrated sulphuric acid hydrolyzed cells of *Hgm. borinquense* strain E3 grown in NSM supplemented with SCB hydrolysate; (A) before soxhlet extraction and (B) after soxhlet extraction. Inset showing comparison of spectrophotometric scans of concentrated acid hydrolyzed standard PHB and polymer obtained from SCB hydrolysate.

TABLE S1: Physico-chemical characterization of the sugarcane bagasse (SCB).

**Sr. no.**	**Characteristic**	**SCB**
**A**	**Physical characteristics**	
**1.**	Colour	Greenish Brown
**2**	Odour	Sweet
**3.**	Texure	
	(a) Procured form	Long fibrous
	(b) Pulverized form	Fine fibrous powder
**4.**	TS (%)	94.3 ± 0.14
**5.**	VS (%)	92.7 ± 0.14
**B**	**Chemical characteristics**	**SCB**
**1.**	CHNS analysis	
	(a) Nitrogen (%)	0.265 ±0.02
	(b) Carbon (%)	43.455 ± 1.2
	(c) Hydrogen (%)	6.095 ±0.27
	(d) Sulphur (%)	0.3225 ± 0.12
	(e) C/N ratio (%)	165.83 ± 8.0
		**SCB hydrolysate**
**2.**	COD (g/Kg)	1.18 ± 0.05
**3.**	Total carbohydrates (g/L)	12.64 ± 0.7
**4.**	TKN (g/L)	0.7
**5.**	C/N ratio (%)	18.05

g/L gram per liter; % percentage
